# Cardiovascular medication seems to promote recovery of autonomic dysfunction after stroke

**DOI:** 10.1007/s00415-022-11204-w

**Published:** 2022-06-12

**Authors:** Ruihao Wang, Martin Köhrmann, Rainer Kollmar, Julia Koehn, Stefan Schwab, Bernd Kallmünzer, Max J. Hilz

**Affiliations:** 1 Department of Neurology, University of Erlangen-Nuremberg, Schwabachanlage 6, 91054 Erlangen, Germany; 2 Department of Neurology, University Hospital Essen, Essen, Germany; 3 Department of Neurology, General Hospital Darmstadt, Darmstadt, Germany; 4 Icahn School of Medicine at Mount Sinai, New York, NY USA

**Keywords:** Ischemic stroke, Autonomic dysfunction, Baroreflex sensitivity, Cardiovascular medication

## Abstract

**Background:**

Stroke may compromise cardiovascular–autonomic modulation (CAM). The longitudinal post-stroke CAM alterations remain unclear as previous studies excluded patients with cardiovascular medication. This study evaluated whether CAM dysfunction improves after several months in patients under typical clinical conditions, i.e., without excluding patients with cardiovascular medication.

**Methods:**

In 82 ischemic stroke patients [33 women, 64.9 ± 8.9 years, NIHSS-scores 2 (interquartile range 1–5)], we evaluated the applications of cardiovascular medication before stroke, during autonomic tests performed within 1 week, 3 and 6 months after stroke onset. We determined resting RR intervals (RRI), systolic, diastolic blood pressures (BPsys), respiration, parameters reflecting total CAM [RRI-standard deviation (RRI-SD), RRI-total powers], sympathetic [RRI-low-frequency powers (RRI-LF), BPsys-LF powers] and parasympathetic CAM [RMSSD, RRI-high-frequency powers (RRI-HF powers)], and baroreflex sensitivity. ANOVA or Friedman tests with post hoc analyses compared patient data with data of 30 healthy controls, significance was assumed for *P* < 0.05.

**Results:**

More patients had antihypertensive medication after than before stroke. First-week CAM testing showed lower RRIs, RMSSD, RRI-SDs, RRI-total powers, RRI-HF powers, and baroreflex sensitivity, but higher BPsys-LF powers in patients than controls. After 3 and 6 months, patients had significantly higher RRIs, RRI-SDs, RRI-total powers, RMSSDs, RRI-HF powers, and baroreflex sensitivity, but lower BPsys-LF powers than in the 1st week; RMSSDs and RRI-HF powers no longer differed between patients and controls. However, 6-month values of RRIs, RRI-SDs, and baroreflex sensitivity were again lower in patients than controls.

**Conclusions:**

Even mild strokes compromised cardiovagal modulation and baroreflex sensitivity. After 3 months, CAM had almost completely recovered. Recovery might be related to the mild stroke severity. Presumably, CAM recovery was also promoted by the increased application of cardiovascular medication. Yet, slight CAM dysfunction after 6 months suggests continuing autonomic vulnerability.

**Supplementary Information:**

The online version contains supplementary material available at 10.1007/s00415-022-11204-w.

## Introduction

Ischemic stroke may induce functional or structural alterations within the central autonomic network (CAN) that compromise cardiovascular autonomic modulation (CAM) [1, 2] which is often characterized by decreased parasympathetic activity and relative sympathetic hyperactivity in the acute [3–7] and chronic phases of stroke [8–10]. The autonomic mechanism may play an important role in the stroke-induced lesioning of the brain–heart axis and subsequent stroke-related cardiac injuries [11, 12].

So far, it is unclear whether altered CAM recovers several months after stroke, since various studies used different patient groups to compare CAM values of the acute and chronic phases after stroke [9] or only reported CAM parameters assessed in the acute phase [3, 4] or in the chronic phase [8, 10].

For example, McLaren et al. compared the CAM in 70 patients 9 months after stroke to CAM in 76 age-matched healthy persons and concluded that CAM was still impaired among the stroke survivors [10]. Dütsch et al. compared CAM in 28 patients 18–43 months after lacunar stroke with CAM in 21 controls and found still reduced cardiovagal modulation in the patients [8]. Xiong and colleagues reported impaired CAM in 34 patients during the 1st week after stroke and in 60 different patients tested 6 months after stroke onset [9]. However, evaluation of different patient groups does not allow to identify the changes in CAM over time.

To our knowledge, only the studies by Korpelainen and coworkers re-evaluated the same patients to assess longitudinal CAM changes after ischemic stroke [6, 7]. In 1996, Korpelainen et al. assessed CAM in 31 stroke patients and found a similar reduction of the overall CAM during the acute phase, after 1 and 6 months [6]. In 1997, the group reported a loss in the circadian CAM in 32 acute stroke patients, but recorded a partial, nocturnal CAM recovery after 6 months, suggesting that impaired CAM may recover to some extent within several months after stroke.

It is noteworthy that both studies had excluded patients who had been on any medication known to affect the autonomic nervous system to ensure that any recorded CAM dysfunction was caused by the stroke and not by any drugs [6, 7]. However, due to preexisting risk factors, most stroke patients need cardiovascular medication, such as antihypertensive or antiarrhythmic drugs that not only reduce the risk of complications and recurrent stroke [13], but may also modify cardiovascular autonomic function [14, 15]. After several months, such medication might have an effect on parameters measuring CAM. We therefore hypothesize that a follow-up study of stroke patients not excluding patients who receive cardiovascular medication will show improvement of CAM after several months. To assess whether and when CAM recovers in ischemic stroke patients, we therefore evaluated CAM parameters in patients with ischemic stroke within the 1st week, and 3 and 6 months after stroke onset without excluding patients with cardiovascular medication.

## Patients and methods

From November 2013 to July 2018, we consecutively enrolled patients with acute ischemic stroke without a history of atrial fibrillation. Further inclusion criteria were: (1) patients with acute cerebral ischemia admitted to our stroke unit, (2) no previous history of AF and no evidence of AF or other cardiac arrhythmias during complete diagnostic workup (including electrocardiogram (ECG) for at least 72 h, transthoracic echocardiography, extracranial und transcranial Doppler sonography, routine laboratory), (3) patients and/or relatives understood the study procedures and were willing and able to participate in the study, (4) minimum CHA2DS2-VASc-Score of 1 (prior to present stroke), (5) age 50 and above. The exclusion criteria of this study were: (1) previously documented episode of atrial fibrillation or flutter, (2) diagnosis of transient ischemic attack, (3) clinically manifest peripheral neuropathy, (4) acute infections, (5) patient and/or relatives were unable to reliably perform the study.

All patients underwent neuroimaging examinations to confirm the infarctions with computed tomography (CT) or magnetic resonance imaging (MRI). The stroke severity was assessed using the National Institutes of Health Stroke Scale (NIHSS) scores.

In all patients, we determined cardiovascular risk factors and compared the percentage of patients on cardiovascular medication before stroke onset, and at the three time points of cardiovascular autonomic recordings, i.e., within the 1st week, and 3 and 6 months after stroke onset. The stroke subtype was classified according to the Trial of Org 10,172 in Acute Stroke Treatment (TOAST) classification [16].

The study has been approved by the Ethics Committee of the University of Erlangen-Nuremberg. Before attending the study, all study participants or their legal representatives signed the written informed consent according to the Declaration of Helsinki.

### Recordings of bio-signals within the 1st week, and 3 and 6 months after stroke onset

At each of the three time points, we monitored heart rate as electrocardiographic RR intervals (RRI), systolic and diastolic blood pressure (BPsys, BPdia), as well as respiratory frequency at supine rest. Measurements were performed between 9 a.m. and 2 p.m. after a 40-min resting period in a reclining armchair, in a quiet room with an ambient temperature of 24 °C and stable humidity [3, 17–19].

We recorded RRIs via a standard three-lead electrocardiogram, and used finger-pulse photoplethysmography (Portapres^®^, Finapres Medical Systems BV, Amsterdam, The Netherlands) to continuously measure beat-to beat Bpsys and Bpdia [17]. Respiratory frequency was monitored with a piezoelectric belt at the lower thorax (at the point of maximal respiratory excursion) [17].

The data were digitized and recorded on a custom-designed data acquisition and analysis system (SUEmpathy, SUESS-Medizin-Technik, Aue, Germany) and stored on a personal computer for off-line analysis [17]. From 5-min recordings without artifacts at rest, we extracted the most stationary 120-s epochs, then calculated mean values and standard deviations (SD) of all signals.

### Calculation of time and frequency domain parameters

To evaluate CAM at rest, we calculated the SD and coefficient of variation (CV) of RRIs, reflecting the total (sympathetic and parasympathetic) cardiac regulation [17, 20]. Moreover, we determined the square root of mean-squared differences of successive RRIs (RMSSD) reflecting parasympathetic cardiac regulation [17, 20].

The values of RRI and BP are modulated by fluctuations that mainly reflect the activity of CAM [17]. We used the trigonometric regressive spectral analysis (TRS) to determine the sympathetically and parasympathetically mediated RRI and BP oscillations [21].

RRI oscillations in the so-called high-frequency band (HF 0.15–0.5 Hz) reflect changes of parasympathetic outflow, while BP oscillations in the HF band are largely a mechanical result of respiration-induced fluctuations in venous return and cardiac stroke volume [17]. BP oscillations in the low-frequency band (LF 0.04–0.15) are mediated by changes in sympathetic outflow, while LF oscillations of RRI contain mainly sympathetic activity but also an unknown amount of parasympathetic oscillations [17, 20].

We quantified the HF and LF components of the bio-signals to determine the powers of sympathetic and parasympathetic influences on RRIs and BP. The magnitude of parasympathetic or sympathetic modulation was calculated as the integral under the power spectral density curves [17, 20].

In addition, we determined the baroreflex sensitivity (BRS). The TRS software selected pairs of LF and HF oscillations of Bpsys and RRI with a coherence above 0.7 which is considered high enough to indicate a stable phase relation—and thus synchronization—between two signals oscillating at this frequency [17–19, 22, 23]. Then, the BRS (ms/mmHg) can be derived as gain values from changes in RRIs (ms) in relation to changes in systolic BP (mmHg) [17, 22, 23].

### CAM comparison between stroke patients and healthy controls

Patient values of the bio-signals and CAM parameters assessed at three time points were compared to the respective values of the same 30 age- and gender-matched healthy controls selected from a pre-existing group of healthy controls whose data had been collected as a reference values of our autonomic laboratory [3] under identical conditions as in the patients.

### Statistical analysis

We used the Kolmogorov–Smirnov test to test for normal data distribution. To compare bio-signals and autonomic parameters of patients between the three time points after stroke onset and with values of healthy controls, we performed analyses of variance (ANOVAs) for repeated measurements (general linear model) for normally distributed data. We used “testing time points” (testing within the 1st week, and 3 and 6 months after stroke onset) as within-subject factor and “participants” (patients and controls) as between-subject factor. Suitability of the ANOVA was assessed by Mauchly’s test of sphericity. In case of violation of the sphericity assumption, the Greenhouse–Geisser correction was employed. In case of significant ANOVA results, we performed post hoc analyses using the paired Student’s *t* test to compare within-subject values assessed at the three time points, and *t* tests for independent samples to compare patient data at each of the three time points with the respective data of the healthy controls.

If data were not normally distributed, we performed Friedman tests to compare the values sampled at the three time points, and Wilcoxon tests for paired samples to compare within-subject values assessed at two of the three time points. The Mann–Whitney *U* Test was applied to compare patient data at each of the three time points with the respective data of the healthy controls.

To compare the number of patients on cardiovascular medication before hospital admission, and at the three time points of testing, we performed the Cochran’s *Q* tests for data assessed at the four time points with post hoc McNemar test for data at the two of the four time points. To compare the dosages, the cardiovascular medication before hospital admission and at the three time points of testing, we performed Friedman tests for dosages at the four time points and Wilcoxon tests for dosages at two of the four time points. Dosages of converting enzyme inhibitors or angiotensin II-receptor blockers, beta-blockers calcium channel blockers, and diuretics were expressed as the equivalent dosages of ramipril, bisoprolol, amlodipine, and torsemide, respectively, according to the guidelines of the European Society of Cardiology for the diagnosis and treatment of heart failure [24, 25].

Normally distributed data were expressed as mean ± standard deviation (SD), and not normally distributed data including NIHSS values were expressed as median with interquartile range (IQR). Significance was set at *P* < 0.05. For statistical analysis, we used IBM SPSS Statistics Version 24 (Armonk, NY, USA).

## Results

### Demographic data of study participants

82 patients with acute ischemic stroke [33 women and 49 men, mean age 64.9 ± 8.9 years] participated in our study. Upon hospital admission, their NIHSS values ranged from 0 to 20 with a median NIHSS value of 2 [IQR 1–5]. The median intervals between stroke onset and the three CAM evaluations were 4 days (IQR 3–6 days), 96 days (IQR 92–100 days), and 186 days (IQR 183–190 days).

Age and gender distribution of the stroke patients did not differ from that of the 30 healthy controls (17 women and 13 men, *P* = 0.122; mean age 63.3 ± 6.8 years, *P* = 0.368).

### Stroke risk factors and subtypes, and cardiovascular medication before and after stroke

Arterial hypertension, diabetes mellitus, dyslipidemia, and nicotine consumption were prevalent in 81.7% (67/82), 15.9% (13/82), 50.0% (41/82), and 28.1% (23/82) of patients, respectively (Supplementary Table 1). 12 patients had previous history of ischemic stroke, and no patient had peripheral neuropathy. 51 patients received cranial MRT examinations, and 31 patients had head CT examinations; 42 patients had infarctions in the territory of the middle and anterior cerebral arteries, and 40 patients had infarctions in the territory of the vertebrobasilar arteries. According to the TOAST classification, 23 (28.0%) patients had large artery atherosclerotic (LAA) stroke, 6 (7.3%) patients had cardioembolic stroke, 31 (37.8%) patients had stroke due to small-vessel occlusion (SVO), 5 (6.1%) patients had stroke due to other reasons, and 17 (20.7%) patients had stroke of undetermined etiology (Supplementary Table 1). Nine (10.8%) patients had acute large vessel occlusion. Table 1 shows the medication of the 82 patients before stroke and during the first, second, and third CAM assessment. Significantly more patients were on angiotensin-converting enzyme inhibitors (ACEIs) or angiotensin II-receptor blockers (ARBs), calcium channel blockers (CCBs), and diuretics, while only a slightly larger number of patients were on beta-blockers during the 1st week after stroke than before stroke. During the 3- and 6-months follow-up assessments, these 1st week numbers had not changed significantly (Table 1). Dosages of ACEI/ARB, CCB, and diuretics were also significantly higher within 1 week and 3 and 6 months after stroke compared with the respective dosages before stroke (Supplementary Table 2). Dosages of beta-blockers were significantly higher only within the 1st week and 3 months after stroke onset, but not 6 months after stroke compared with values before stroke (Supplementary Table 2). Dosages of ACEI/ARB and beta-blockers showed a slight and non-significant decrease 3 and 6 months after stroke onset compared with dosages within 1 week after stroke onset (Supplementary Table 2).

**Table 1:** Medications before admission, within 1 week and 3 and 6 months after stroke onset among the 82 patients with ischemic stroke

Medications	82 patients with ischemic stroke				*P* values			
	Before admission	Within 1 week (I)	After 3 months (II)	After 6 months (III)	Cochran’s Q test	P1^a^ (I vs. II)	P2^a^ (I vs. III)	P3^a^ (II vs III)
Antiplatelet therapy	21/82 (25.6%)	81/82 (98.8%) ***	78/82 (95.1%) ***	72/82 (87.8%) ***	** < 0.001**	0.250	**0.012**	0.070
Statins	22/82 (26.8%)	82/82 (100%) ***	72/82 (87.8%) ***	67/82 (81.7%) ***	** < 0.001**	**0.002**	** < 0.001**	0.063
ACEI/ARB	40/82 (48.8%)	63/82 (76.8%) ***	64/82 (78.1%) ***	59/82 (71.9%) **	** < 0.001**	1.000	0.424	0.125
Beta-blockers	23/82 (28.1%)	30/82 (36.6%)	30/82 (36.6%)	25/82 (30.5%)	**0.038**	1.000	0.227	0.063
CCB	17/82 (20.7%)	27/82 (32.9%) **	25/82 (30.5%)	25/82 (30.5%)	**0.031**	0.774	0.791	1.000
Diuretics	14/82 (17.1%)	32/82 (39.0%) ***	25/82 (30.5%) *	25/82 (30.5%) *	** < 0.001**	0.143	0.138	1.000

Values of significant differences between medications before admission and the following three time points (within 1 week, 3-months follow-up, 6-months follow-up) are expressed ins ***bold and italic***, with *indicating *P* < 0.05, **indicating *P* < 0.01, and ***indicating *P* < 0.001

*ACEI* angiotensin-converting-enzyme inhibitor, *ARB* angiotensin II-receptor blocker, *CCB* calcium channel blocker

^a^Post hoc McNemar test. P1, within 1 week vs. 3 months. P2, within 1 week vs. 6 months, P3, 3 months vs. 6 months

After discharge from our stroke unit, 80.5% (66/82) of the patients were admitted to a rehabilitation center.

### Bio-signals in stroke patients and healthy controls

**Fig. 1:**
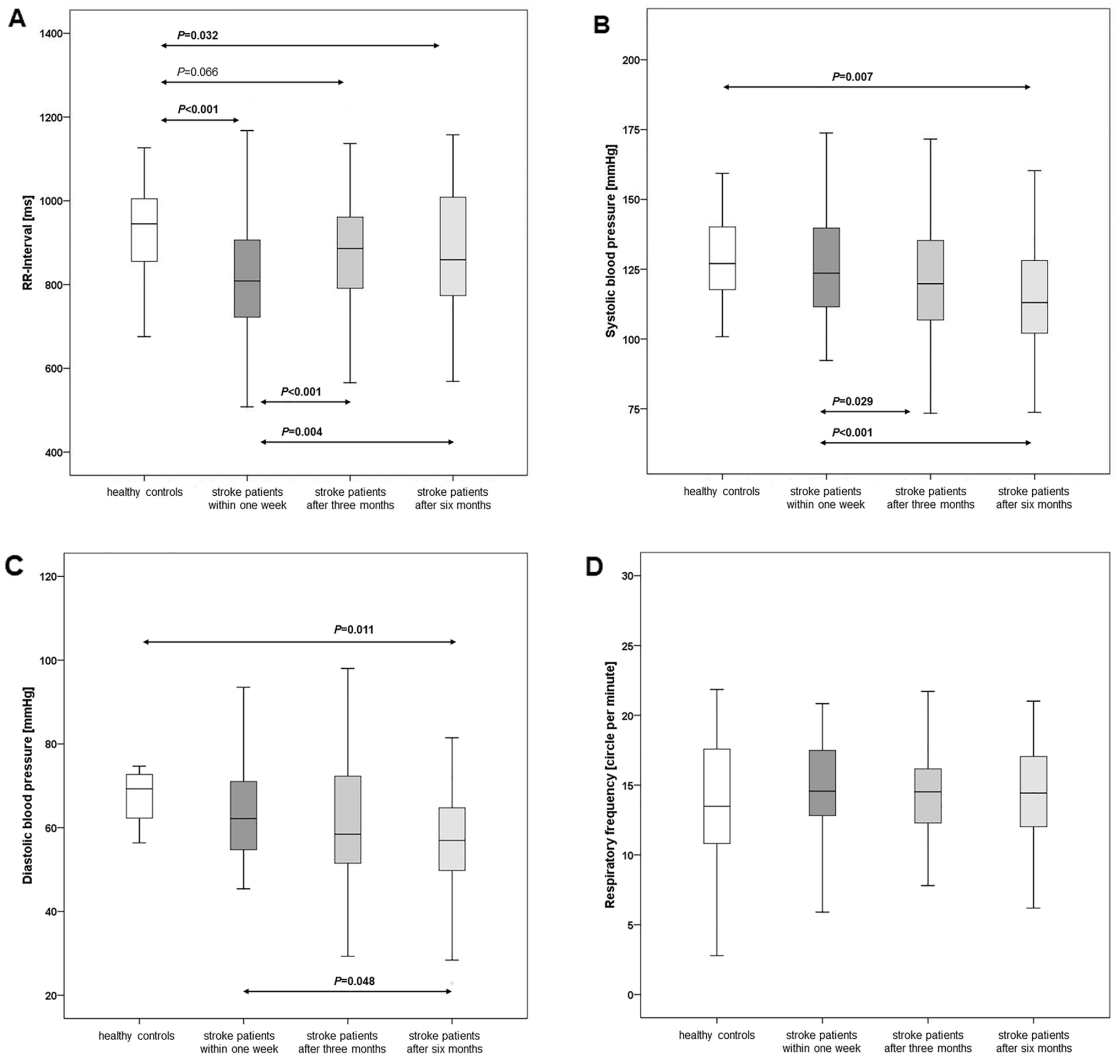
RR interval, systolic and diastolic blood pressure, and respiratory frequency in 30 healthy controls and 82 stroke patients assessed within 1 week and 3 and 6 months after stroke onset. Data are presented as box plots. The line in the middle of the box represents the median (50th percentile), the top of the box represents the upper quartile (75th percentile), the bottom of the box represents the lower quartile (25th percentile), and the end of the whiskers represents the highest and lowest values that are not extreme values or outliers

ANOVA showed significant differences in RRI values between patients and controls and among patients at the three time points (Table 2). In the patients, RRIs assessed within the 1st week after stroke were significantly lower (826.1 ± 146.0 ms) than in the 30 controls (935.2 ± 122.0 ms, *P* < 0.001; Table 2, Fig. 1). However, RRIs had significantly re-increased after 3 months (885.1 ± 127.7 ms, *P* < 0.001; Table 2, Fig. 1) and 6 months (876.0 ± 142.2 ms, *P* = 0.004). After 3 months, patient RRIs no longer differed from RRIs of controls, while patient RRIs after 6 months were again slightly though significantly lower than control values (Table 2, Fig. 1).

**Table 2:** Bio-signals and time-domain parameters in 30 healthy controls and 82 patients with ischemic stroke assessed within 1 week and 3 and 6 months after stroke onset

Bio-signals and time-domain parameters	30 healthy controls	82 patients with ischemic stroke			*P* values			
		Within 1 week (I)	After 3 months (II)	After 6 months (III)	ANOVA or Friedman test	P1 (I vs. II)	P2 (I vs. III)	P3 (II vs III)
RRI [ms]	935.2 ± 122.0	*826.1* ± 146.0***	885.1 ± 127.7	*876.0* ± 142.2*	** < 0.001**^a^	** < 0.001**^c^	**0.004**^c^	0.384^c^
BPsys [mmHg]	127.7 ± 15.6	127.4 ± 23.4	120.2 ± 20.4	*115.6* ± 20.3**	**0.001**^a^	**0.029**^**c**^	** < 0.001**^c^	0.141^c^
BPdia [mmHg]	66.5 ± 8.3	62.9 ± 11.4	61.3 ± 15.0	*59.0* ± 13.8*	0.182^a^	0.340^c^	**0.048**^**c**^	0.374^c^
RESP [cpm]	13.8 ± 4.4	14.9 ± 3.4	14.1 ± 3.3	14.3 ± 3.3	0.245^a^	0.102^c^	0.275^c^	0.638^c^
RRI-SD [ms]	24.0 ± 8.9	*18.7* ± 11.7**	23.2 ± 14.2	*21.6* ± 12.9*	**0.035**^**b**^	**0.004**^d^	**0.034**^d^	0.393^d^
RRI-CV [%]	2.5 ± 0.9	*2.2* ± 1.1*	2.6 ± 1.5	2.4 ± 1.4	0.355^b^	**0.033**^**d**^	0.136^d^	0.409^d^
RMSSD [ms]	19.1 ± 9.9	*15.2* ± 14.4**	19.7 ± 15.5	18.4 ± 13.9	**0.006**^b^	**0.001**^d^	**0.013**^d^	0.227^d^

Data were expressed as mean ± standard deviations. ^a^Repeated measurements of ANOVA. ^b^Friedman test. ^c^Paired *t *test. ^d^Wilcoxon-signed ranks test. Data of healthy controls were compared to data of stroke patients using the Student’s *t* test for normally distributed data and the Mann–-Whitney *U* test for not normally distributed data. Values of significant differences between patients and controls were expressed in *bold and italic*, with * indicating *P* < 0.05, ** indicating *P* < 0.01, and *** indicating *P* < 0.001. P1, within 1e week vs. after 3 months. P2, within 1 week vs. after 6 months, P3, after 3 months vs. after 6 months

*RRI* RR interval, *Bpsys* systolic blood pressure, *Bpdia* diastolic pressure, *RESP* respiratory frequency, *RRI-SD* standard deviation of RRI, *RRI-CV* coefficient of variation of RRI, *RMSSD* square root of the mean-squared differences of successive RRIs

The 1st week assessment showed BPsys and BPdia values in patients (127.3 ± 23.4 mmHg; 62.9 ± 11.4 mmHg) similar to those of controls (127.7 ± 15.6 mmHg; 66.5 ± 8.3 mmHg; Table 2, Fig. 1). After 3 months, patients’ BPsys values (120.2 ± 20.4 mmHg, *P* = 0.029), but not BPdia values (61.3 ± 15.0 mmHg), had significantly decreased. After 6 months, both BPsys (115.6 ± 20.3 mmHg; *P* < 0.001) and BPdia (59.0 ± 13.8 mmHg) had decreased and were even lower than in the controls (Table 2, Fig. 1).

Respiratory frequency did not differ between patients and controls or among patients at the three time points (Table 2, Fig. 1).

### CAM time-domain parameters in stroke patients and healthy controls

**Fig. 2:**
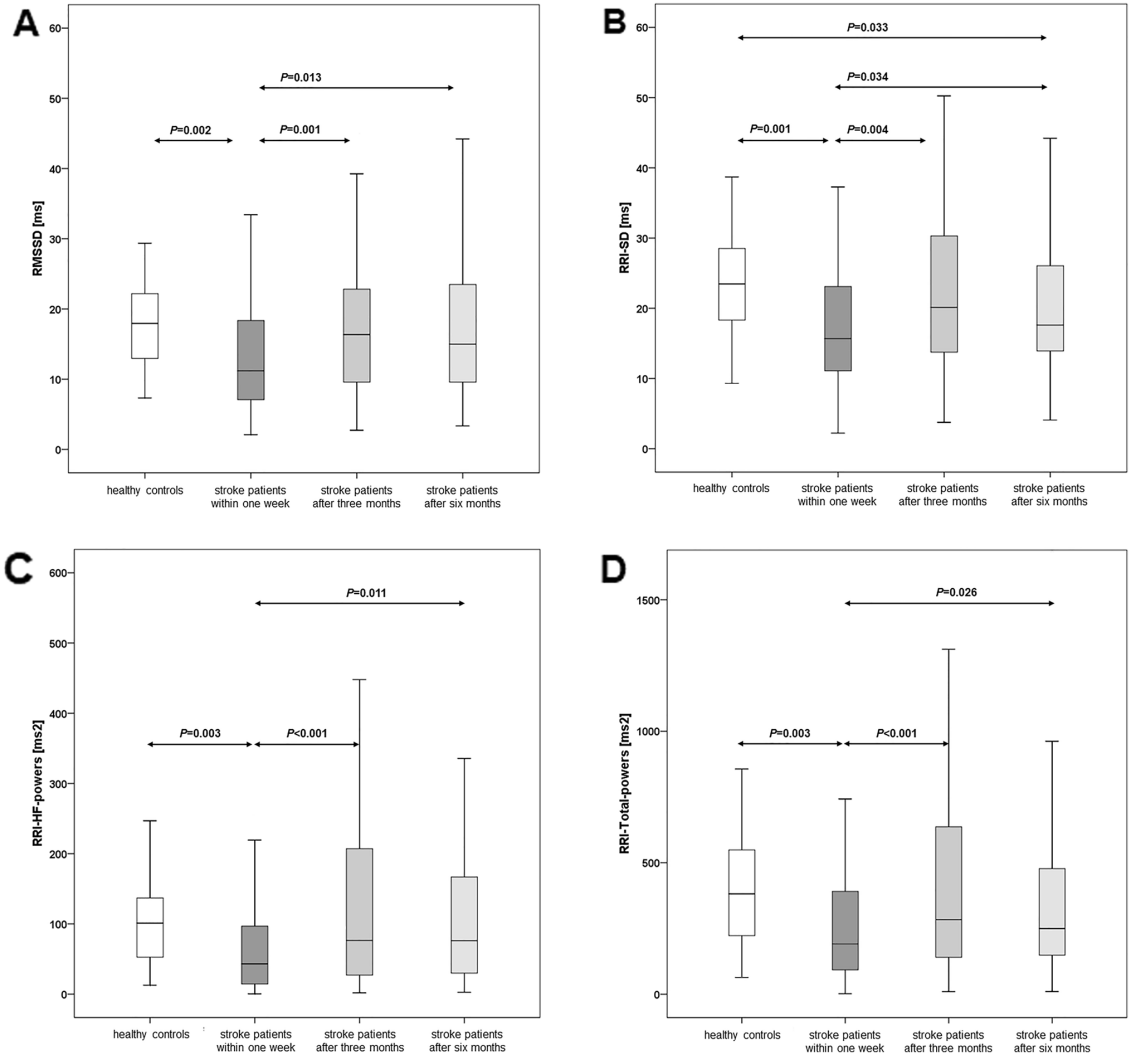
RMSSD, RRI-SD, RRI-HF powers, and RRI-Total powers in 30 healthy controls and 82 stroke patients assessed within 1 week and 3 and 6 months after stroke onset. The line in the middle of the box represents the median (50th percentile), the top of the box represents the upper quartile (75th percentile), the bottom of the box represents the lower quartile (25th percentile), and the end of the whiskers represents the highest and lowest values that are not extreme values or outliers

At the first assessment, patients’ values were significantly lower than in controls for RRI-SD (18.7 ± 11.7 ms vs. 24.0 ± 8.9 ms, *P* < 0.01), RRI-CV (2.2 ± 1.1% vs. 2.5 ± 0.9%, *P* < 0.05), and RMSSD (15.2 ± 14.4 ms vs. 19.1 ± 9.9 ms, *P* < 0.01; Table 2, Fig. 1). After 3 months, RRI-SD, RRI-CV, and RMSSD had significantly increased and no longer differed from control values. After 6 months, RRI-SD values again were slightly, though significantly, lower than in controls, while RRI-CV and RMSSD,values again remained similar to control values (Table 2, Fig. 2).

### CAM frequency-domain parameters in stroke patients and healthy controls

Similar to the time-domain parameters, patient values of the first assessment were significantly lower than control values for RRI-LF powers (206.1 ± 231.3 ms^2^ vs. 294.0 ± 218.8 ms^2^, *P* < 0.01), RRI-HF powers (91.0 ± 139.2 ms^2^ vs. 118.5 ± 124.2 ms^2^, *P* < 0.01), and RRI-total powers (297.1 ± 321.9 ms^2^ vs. 412.5 ± 292.2 ms^2^, *P* < 0.01). Again, 3- and 6-month values of patients had significantly increased and no longer differed from the respective control values (Table 3, Fig. 2).

**Table 3:** Frequency-domain parameters in 30 healthy controls and 82 patients with ischemic stroke assessed within 1 week and 3 and 6 months after stroke onset

Frequency-domain parameters	30 healthy controls	82 patients with ischemic stroke			*P* values			
		Within 1 week (I)	After 3 months (II)	After 6 months (III)	Friedman test	P1^a^ (I vs. II)	P2^a^ (I vs. III)	P3^a^ (II vs III)
RRI-LF powers [ms^2^]	294.0 ± 218.8	*206.1* ± 231.3**	398.8 ± 610.7	313.9 ± 600.0	0.081	**0.002**	0.272	0.109
RRI-HF powers [ms^2^]	118.5 ± 124.5	*91.0* ± 139.2**	170.2 ± 228.5	116.8 ± 119.8	**0.00**	** < 0.001**	**0.011**	0.129
RRI-Total powers [ms^2^]	412.5 ± 292.2	*297.1* ± 321.9**	568.9 ± 747.8	430.8 ± 651.8	**0.029**	** < 0.001**	**0.026**	0.071
BPsys-LF powers [mmHg^2^]	7.7 ± 7.2	*15.8* ± 15.1***	11.1 ± 10.1	11.5 ± 10.4*	**0.003**	**0.005**	**0.019**	0.134
BPsys-HF powers [mmHg^2^]	1.8 ± 2.0	*5.6* ± 5.8***	*4.4* ± 5.2**	*4.9* ± 8.0***	**0.030**	**0.006**	0.053	0.625
BRS [ms/mmHg]	6.8 ± 3.9	*3.8* ± 3.1***	5.5 ± 3.8	*4.8* ± 2.9**	** < 0.001**	** < 0.001**	** < 0.001**	**0.033**

Data were expressed as mean ± standard deviations. ^a^Wilcoxon signed ranks test. Comparisons between the healthy controls and stroke patients were performed using the Mann–Whitney *U* test. Values of significant differences between patients and controls are expressed in bold and italic, with *indicating *P* < 0.05, **indicating P < 0.01, and ***indicating *P* < 0.001. P1, assessment within 1 week vs. after 3 months. P2, assessment within 1e week vs. after 6 months, P3, assessment after 3 months vs. after 6 months

*RRI* RR interval, *HF* high frequency, *LF* low frequency, *BPsys* systolic blood pressure, *BRS* baroreflex sensitivity

**Fig. 3:**
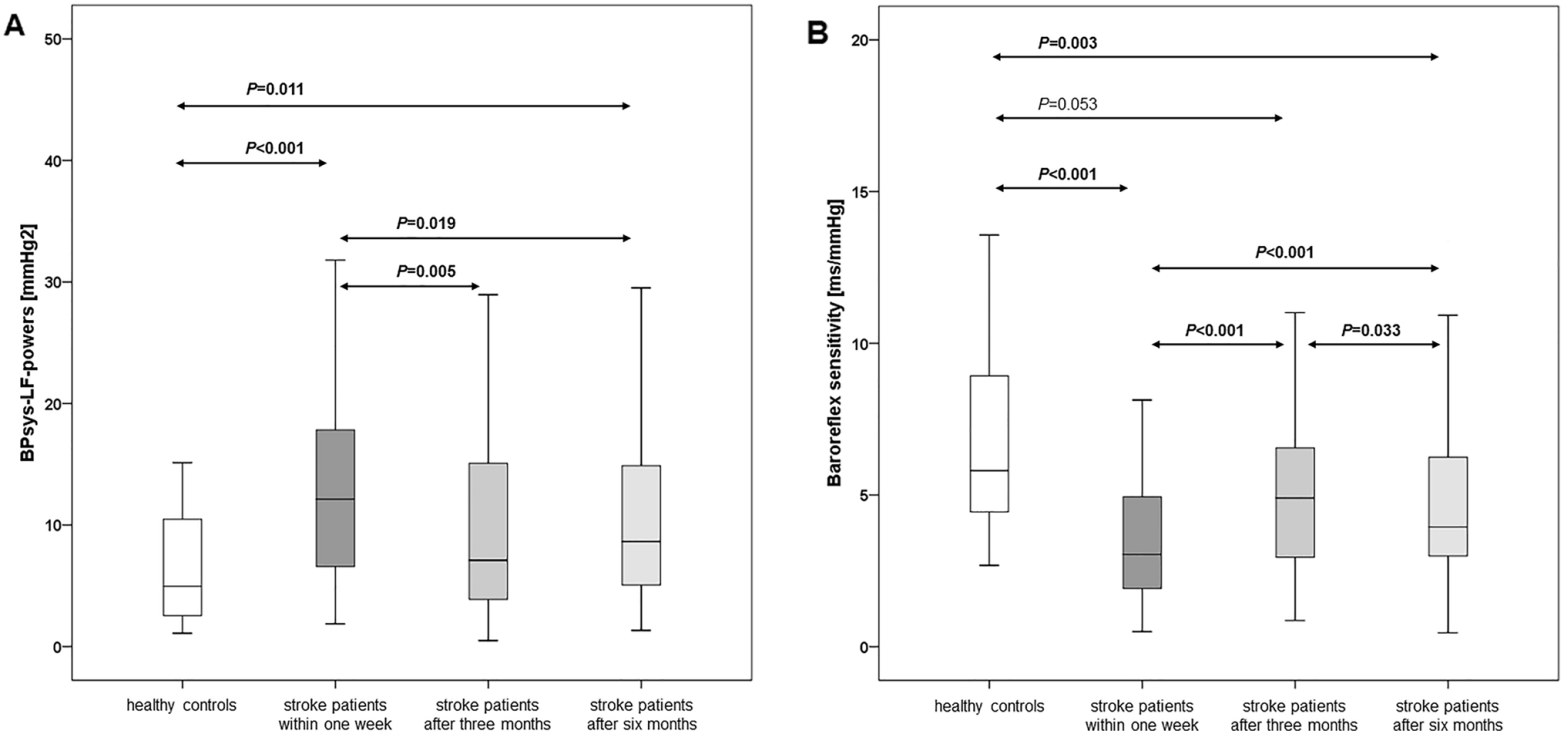
BPsys-LF powers and baroreflex sensitivity in 30 healthy controls and 82 stroke patients assessed within 1 week and 3 and 6 months after stroke onset. The line in the middle of the box represents the median (50th percentile), the top of the box represents the upper quartile (75th percentile), the bottom of the box represents the lower quartile (25th percentile), and the end of the whiskers represents the highest and lowest values that are not extreme values or outliers

In contrast, at the first assessment, sympathetically mediated BPsys-LF powers were significantly higher in patients (15.8 ± 15.1 mmHg^2^) than were the control values (7.7 ± 7.2 mmHg^2^; *P* < 0.001, Table 3, Fig. 3). Yet, BPsys-LF powers had significantly decreased after 3 (11.1 ± 10.2 mmHg^2^, *P* = 0.005) and 6 months (11.5 ± 10.4 mmHg^2^, *P* = 0.019). Three-month values of BPsys-LF powers no longer differed from control values, while 6-months BPsys-LF powers were again slightly higher in patients than in controls (*P* = 0.011, Table 3, Fig. 3).

The mechanically mediated BPsys-HF powers of patients (5.6 ± 5.8 mmHg^2^) initially also were significantly higher than in controls (1.8 ± 2.0 mmHg^2^, *P* < 0.001, Table 3), but had significantly decreased after 3 months (4.4 ± 5.2 mmHg^2^, *P* = 0.006) while the reduction after 6 months was not quite significant (4.9 ± 8.0 mmHg^2^, *P* = 0.053), and 3- and 6-month BPsys-HF powers were still significantly higher than in controls (Table 3).

At the first assessment, BRS values of patients also were lower than in controls (3.8 ± 3.1 ms/mmHg vs. 6.8 ± 3.9 ms/mmHg, *P* < 0.001; Table 3, Fig. 3). Yet, BRS values had significantly increased after 3 (5.5 ± 3.8 ms/mmHg, *P* < 0.001) and 6 months (4.8 ± 2.9 ms/mmHg, *P* < 0.001). Three-month BRS values no longer differed from BRS values in controls. However, 6-month BRS values were again significantly lower than in controls (Table 3, Fig. 3).

## Discussion

Our study yields four major results and supports conclusions regarding stroke-related cardiovascular autonomic dysfunction and its recovery [1]: even after mild ischemic stroke, there was cardiovascular autonomic dysregulation during the 1st week after stroke onset [2]; cardiovascular autonomic dysregulation at rest recovered almost completely within the first 3 months after stroke onset [3]; surprisingly, autonomic cardiovascular regulation had slightly deteriorated after 6 months [4]; in contrast to the previous follow-up studies by Korpelainen et al. who assessed cardiovascular autonomic regulation in the same patients within 1 week, after 1 and 6 months [6, 7], our patients had received cardiovascular medication throughout the study.

In more detail, our patients had median NIHSS scores of only 2 (IQR 1–5) and thus milder strokes than the patients evaluated by Korpelainen and co-workers in their studies [5–7]. The Finnish patients had an average stroke severity of 40 on the Scandinavian Stroke Scale [6] which converts to an NIHSS score of approximatley 5 [26].

Still, autonomic testing of our rather mild stroke patients several days after stroke onset (median interval 4 days, IQR 3–6 days) unveiled CAM dysfunction similar to that reported in previous stroke studies [3–6]. Compared to our control group, the patients had lower RRIs, i.e., higher heart rates, lower RRI-SD values and RRI-total powers, i.e., lower overall CAM, reduced cardiovagal modulation as shown by the lower RMSSD values and RRI-HF powers, but increased sympathetic vascular modulation as shown by the higher BPsys-LF powers in patients than controls. Furthermore, baroreflex sensitivity was lower in patients than controls.

The altered CAM has clinical and prognostic implications [3, 11, 12]. Reduced cardiovagal modulation with a shift toward sympathetic predominance and a compromised baroreflex sensitivity are associated with an increased risk of poor outcome [6, 27], secondary brain injuries [28], and increased mortality rates [1, 6, 11]. Particularly reduced cardiovagal modulation is associated with an increased risk of recurrent strokes, as shown by Guan et al. [29].

The first autonomic evaluation after a median interval of 4 days (IQR 3–6 days) since stroke onset also showed that CAM dysregulation may persist beyond the first 72 h after stroke onset even after mild strokes. In contrast, Kallmünzer et al. observed heart rate recovery in stroke patients 72 h after hospital admission, suggesting normalization of cardiovagal modulation already within a few days after stroke onset [30]. Similarly, Sposato et al. assume that the autonomic dysfunction after stroke onset will start to fade several days after stroke [31]. However, previous studies support the conception that the site and size of the cerebral lesions and the involvement of areas contributing to the central autonomic network will determine the extent and most likely the persistence of autonomic changes upon stroke [1, 2, 11, 12, 32, 33]. More than 70% of our patients had stroke not due to large artery atherosclerosis, and most of our patients did not have acute large-vessel occlusion. Compared with previous studies in which the enrolled stroke patients had more severe neurological impairment and the localizations were more often also territorial and cortical [3, 4, 6, 9, 34, 35], our data suggest that even mild stroke may induce cardiovascular autonomic dysfunction.

Yet, our second major finding, the almost complete recovery of resting CAM within the first 3 months after stroke onset, suggests that stroke severity also impacts the duration of autonomic derangements. In their more severely afflicted stroke patients, Korpelainen et al. did not observe a significant recovery of autonomic modulation even after 6 months [6]. In contrast, RRIs and all autonomic parameters of our patients, after 3 months, no longer differed from our healthy control values. The recovery of the overall CAM, particularly the re-increased cardiovagal modulation and baroreflex sensitivity, the lowered heart rates with attenuated sympathetic activity are all associated with improved cardiovascular prognosis [22, 36, 37] and a lowered risk of recurrent ischemic events [29].

Our third main finding, the slight CAM deterioration after 6 months, suggests that the central CAM regulation was not yet quite stable, although the patients had only mild strokes. The patients still had increased higher sympathetically mediated BP modulation, as evidenced by their higher BPsys-LF powers than in the controls, and their baroreflex function was altered as shown by lower 6-months BRS values in the patients than the controls (Table 3). Moreover, the patients again had higher heart rates and slightly lower RRI-SDs than the controls (Table 2). Yet, RRI-CV values and RRI-total powers again did not differ from control values suggesting that the decrease in the overall CAM was only minor after 6 months. The finding of lower baroreflex sensitivity, higher sympathetic modulation, and slightly lower overall cardiac autonomic modulation in the patients 6 months after stroke than in controls is in line with previous studies, which showed cardiovascular autonomic dysfunction even in the chronic phase after stroke [4, 9, 10, 38].

For ethical reasons, we assessed CAM in our stroke patients only at supine rest and avoided autonomic challenge maneuvers. Yet, our previous studies of patients with a history of mild traumatic brain injury showed that autonomic instability with minor, subclinical cardiovascular dysregulation upon challenge may persist even years after the initial trauma [39]. Six months after stroke, our patients quite likely tried to steadily return to their daily life activities and thus might have been facing more stressful challenges than only 12 weeks after the stroke onset, when patients assumingly still rested more and were better shielded against daily life challenges. Moreover, perhaps due to reduced compliance, slightly less patients were on statins and antihypertensive drugs 6 months after stroke onset than during the initial and the 3-month evaluations (Table 1) which might have added to a somewhat higher instability of cardiovascular autonomic modulation. Also, 6 months after stroke, the slight decrease in individual dosages of ACEI/ARB and beta-blockers (Supplementary Table 2) might partly explain the minor CAM deterioration.

However, the overall change in autonomic parameters, 3 and 6 months after stroke onset, provides convincing evidence that the centrally mediated CAM improved significantly at rest and no longer showed any clinically significant difference from the autonomic modulation in the healthy age-matched controls.

While Korpelainen et al. did not see a significant CAM recovery after 1 and 6 months [6], the good CAM recovery of our patients may be due to their lower stroke severity that is associated with a better neuroplasticity [40] and thus higher neuro-rehabilitation potential than in more severe strokes. Yet, the major difference between the study of Korpelainen et al. [6] and ours is their exclusion of patients on medication possibly affecting CAM [6], while we had included patients on cardiovascular medication and further adjusted medication upon hospital admission and after 3 and 6 months as needed (Table 1). Significantly more number of our patients were on antihypertensive medication while hospitalized as well as 3 and 6 months after than before stroke (Table 1). The beneficial effects of the antihypertensive medications were already manifest within 1 week after stroke onset, when BP values no longer differed between the patients and controls, although 67/82 patients had a history of arterial hypertension (Table 2). Beta-blockers certainly contributed to the patient group’s decrease in heart rate that was evident after 3 months [41]. The increased use of antihypertensives added to the recovery of baroreflex sensitivity, since the shift of heart rate and blood pressure to lower values on the sigmoid baroreflex curve increases the baroreflex gain in hypertensive patients [14] and over time contributes to baroreflex resetting and improved baroreflex output, very likely via a change in the central command, i.e., via central adjustment with reduced sympathetic and augmented parasympathetic baroreflex responses [15].

Numerous clinical studies demonstrated these beneficial effects of antihypertensives on cardiovascular autonomic regulation [42]. ACE inhibitors, ARBs, or CCBs attenuate the sympathetic tone, augment cardiovagal modulation, and increase baroreflex sensitivity [42–44]. Therefore, we assume that the increased use of cardiovascular medication in our patients may have contributed to CAM recovery with lowered heart rate, re-increased parasympathetic modulation, and baroreflex sensitivity as well as attenuated sympathetic predominance, already within 3 months after stroke onset. As outlined above, these changes are associated with a better prognosis, lower risk of stroke recurrencies, and reduced risk of sudden death [22, 29, 36, 37, 45]. Morever, even the increase in statin treatment may have contributed to the CAM improvement because statins may reduce sympathetic outflow [46].

### Limitations of our study

Since our patients had rather mild strokes, the finding of an almost complete recovery of autonomic modulation at rest cannot be generalized. Several studies showed long-lasting autonomic dysregulation after stroke [1, 8–10], which was quite likely due to a higher stroke severity or a more severe involvement of central autonomic network structures that are essential for cardiovascular control [2]. Moreover, our results do not allow any conclusion regarding autonomic adjustment to challenge. In fact, our 6-months data as well as the aforementioned subtle CAM dysregulation seen in patients years after a mild traumatic brain injury [18, 19, 39] suggest that there might still be autonomic dysregulation during autonomic challenges beyond 6 months, even after mild strokes. We have not compared the data of our patients on cardiovascular medication with a group of patients that has similar and thus comparable age and gender distribution, severity, locations, and types of stroke, as well as pre-existing risk factors, particularly cardiac conditions, but needed no cardiovascular medication, as it is difficult to find such a control group. The absence of such a control group without cardiovascular medication weakens our conclusion. Still, we can conclude that patients who have cardiovascular risk factors and require a therapeutic adjustment of cardiovascular drugs show improvement of cardiovascular autonomic function while receiving this cardiovascular therapy. Another limitation of the study is the fact that we did not compare the stroke patients to a control group with the same pre-existing risk factors as in the patient group but without previous strokes. While it would be desirable to have such a control group, it is—in our experience—rather difficult to establish a control group that has the same baseline characteristics as those of the stroke patients prior to their strokes. Such a control group would need to have not only the same age and gender distribution, weight, and body mass index, but also the same spectrum, history, duration, and treatment regimens of pre-existing conditions and their sequelae as we found them in the stroke patients. Due to the difficulties creating such a control group, we compared the findings of the stroke patients with those in healthy controls. Although we cannot exclude that a control group of healthy persons with similar age and gender distribution very likely has cardiovascular and autonomic parameters that differ from those of the stroke patients already prior to their stroke onset, using healthy controls also has the advantage that their cardiovascular and autonomic parameters can be considered benchmark criteria for a full recovery from any stroke induced cardiovascular autonomic dysregulation.

## Conclusion

In summary, not only the rather mild stroke severity in our patients, but quite likely also the individually adjusted treatment with cardiovascular medication contributed to the full CAM recovery at rest already within the first 3 months after stroke onset. The subtle autonomic dysregulation recorded after 6 months might be due to the patients returning to more daily-life activities and thus an exposure to more autonomic challenges.

Our findings support two conclusions: individualized treatment with antihypertensives and statins seems to support CAM revovery and thus improves the patients’ prognosis. Yet, CAM is still vulnerable 6 months after stroke and might be altered during challenge for a continous period of time. Therefore, follow-up CAM evaluations and individualized treatment adjustments are indicated.

## Supplementary Information

Below is the link to the electronic supplementary material.

Supplementary file1 (DOCX 17 kb)
