# Supplementary material for: Cardiovascular medication seems to promote recovery of autonomic dysfunction after stroke
Source: J Neurol. 2022 Jun 12;269(10):5454–65. doi: 10.1007/s00415-022-11204-w (PMC9467950; doi:10.1007/s00415-022-11204-w)
Supplement: Supplementary file 1 — Supplementary file1 (DOCX 17 kb) [file 415_2022_11204_MOESM1_ESM.docx]

**Supplementary Table 1. Demographic and clinical data of stroke patients and healthy controls**

| Characteristics | 82 patients with ischemic stroke | 30 healthy controls | P VALUES |
| --- | --- | --- | --- |
| Age, mean ± SD [years] | 64.9 ± 8.9 | 63.3 ± 6.8 | 0.368 |
| Women, n (%) | 33 (40.2%) | 17 (56.7%) | 0.122 |
| Pre-mRS, median (IQR) | 0 (0-0) | N.A. | N.A. |
| NIHSS upon admission, median (IQR) | 2 (1-5) | N.A. | N.A. |
| TOAST classification |  |  |  |
| LAA subtype | 23 (28.0%) | N.A. | N.A. |
| CE subtype | 6 (7.3%) | N.A. | N.A. |
| SVO subtype | 31 (37.8%) | N.A. | N.A. |
| Other reasons | 5 (6.1%) | N.A. | N.A. |
| Unknown etiology | 17 (20.7%) | N.A. | N.A. |
| Hypertension, n (%) | 67 (81.7%) | N.A. | N.A. |
| Diabetes mellitus, n (%) | 13 (15.8%) | N.A. | N.A. |
| Dyslipidemia, n (%) | 41 (50%) | N.A. | N.A. |
| Nicotine consumption, n (%) | 23 (28.0%) | N.A. | N.A. |
| Coronary artery disease, n (%) | 10 (12.2%) | N.A. | N.A. |
| Previous history of ischemic stroke or TIA, n (%) | 12 (14.6%) | N.A. | N.A. |
| Acute large vessel occlusion, n (%) | 9 (10.8%) | N.A. | N.A. |
| Acute therapy |  |  |  |
| Intravenous therapy with rtPA, n (%) | 12 (14.6%) | N.A. | N.A. |
| Endovascular mechanical thrombolysis, n (%) | 5 (6.1%) | N.A. | N.A. |

SD, standard deviation; mRS, modified Rankin scale; N.A., not applicable; IQR, interquartile range; NIHSS, National Institutes of Health Stroke Scale; LAA, large artery atherosclerosis; CE, cardio-embolism; SVO, small vessel occlusion; TIA, transient ischemic attack; rtPA, recombinant tissue-type plasminogen activator

**Supplementary table 2. Dosages of the antihypertensive medications before admission, within 1 week and 3 and 6 months after stroke onset among the 82 patients with ischemic stroke**

| Dosages of Medications | 82 patients with ischemic stroke | | | | P values | | | |
| --- | --- | --- | --- | --- | --- | --- | --- | --- |
|  | Before admission | Within 1 week  (I) | After 3 months  (II) | After 6 months  (III) | Freidman test | P1 ^a^  (I vs. II) | P2 ^a^  (I vs. III) | P3 ^a^  (II vs. III) |
| ACEI/ARB, median (IQR) mg/d  equivalent dosage of ramipril | 1.46 (0-5) | ***5 (2.5-10) ****** | ***5 (1.72-10) ****** | ***5 (0-10) ***** | <0.001 | 0.762 | 0.203 | 0.175 |
| 0 mg/d | 42/82 (51.2%) | 19/82 (23.2%) | 18/82 (21.9%) | 23/82 (28.0%) |  |  |  |  |
| (0-5] mg/d | 22/82 (26.8%) | 30/82 (36.6%) | 32/82 (39.0%) | 28/82 (34.1%) |  |  |  |  |
| (5-10] mg/d | 18/82 (21.9%) | 33/82 (40.2%) | 32/82 (39.0%) | 31/82 (37.8%) |  |  |  |  |
| Betablockers, median (IQR) mg/d  equivalent dosage of bisoprolol | 0 (0-1.25) | ***0 (0-2.5) **** | ***0 (0-2.5) **** | 0 (0-1.25) | 0.118 | 0.932 | 0.302 | 0.131 |
| 0mg/d | 59/82 (71.9%) | 52/82 (63.4%) | 52/82 (63.4%) | 57/82 (69.5%) |  |  |  |  |
| (0-5] mg/d | 20 (24.4%) | 27/82 (32.9%) | 27/82 (32.9%) | 23/82 (28.0%) |  |  |  |  |
| (5-10] mg/d | 3/82 (3.7%) | 3/82 (3.7%) | 3/82 (3.7%) | 2/82 (2.4%) |  |  |  |  |
| CCB, median (IQR) mg/d  equivalent dosage of amlodipine | 0 (0-0) | ***0 (0-1) ***** | ***0 (0-1) ***** | ***0 (0-1) ****** | <0.001 | 0.739 | 0.366 | 0.414 |
| 0mg/d | 65/82 (79.3%) | 55/82 (67.1%) | 57/82 (69.5%) | 57/82 (69.5%) |  |  |  |  |
| (0-5] mg/d | 12/82 (14.6%) | 14/82 (17.1%) | 7/82 (8.5%) | 10/82 (12.2%) |  |  |  |  |
| (5-10] mg/d | 5/82 (6.1%) | 13/82 (15.8%) | 18/82 (22.0%) | 15/82 (18.3%) |  |  |  |  |
| Diuretics, median (IQR) mg/d  equivalent dosage of torsemide | 0 (0-0) | ***0 (0-5) ****** | ***0 (0-5) **** | ***0 (0-5) **** | <0.001 | 0.278 | 0.201 | 0.579 |
| 0mg/d | 68/82 (82.9%) | 50/82 (61.0%) | 57/82 (69.5 %) | 57/82 (69.5%) |  |  |  |  |
| (0-5] mg/d | 5/82 (6.1%) | 16/82 (19.5%) | 12/82 (14.6%) | 12/82 (14.6%) |  |  |  |  |
| (5-10] mg/d | 9/82 (11.0%) | 16/82 (19.5%) | 13/82 (15.9%) | 13/82 (15.9%) |  |  |  |  |

a. Wilcoxon signed ranks test. P1, within 1 week vs. 3 months. P2, within 1 week vs. 6 months, P3, 3 months vs. 6 months. Significant differences between dosages of medications before admission and the following three time points (within 1 week, 3-month follow-up, 6-month follow-up) are expressed in ***bold and italic***, with * indicating P<0.05, ** indicating P<0.01, and *** indicating P<0.001

ACEI=angiotensin-converting enzyme inhibitor, ARB=angiotensin II-receptor blocker, CCB=calcium channel blocker, IQR= interquartile range

Dosages of the medications were expressed as median (IQR). In patients who did not take these medications, dosages were defined as 0mg/d. Dosages of ACEI/ARB were expressed as equivalent dosage of ramipril (mg/day), dosages of beta-blockers were expressed as equivalent dosage of bisoprolol (mg/day), dosages of CCB were expressed as equivalent dosage of amlodipine (mg/day), dosages of diuretics were expressed as equivalent dosage of torsemide (mg/day)
